# Vegetarian diets and risk of all-cause mortality in a population-based prospective study in the United States

**DOI:** 10.1186/s41043-023-00460-9

**Published:** 2023-11-23

**Authors:** Keeley Blackie, Gerd Bobe, Yumie Takata

**Affiliations:** 1 Health Promotion and Health Behavior Program, College of Health, Oregon State University, Corvallis, OR USA; 2 Linus Pauling Institute, Oregon State University, Corvallis, OR USA; 3 Nutrition Program, College of Health, Oregon State University, 103 Milam Hall, Corvallis, OR USA

**Keywords:** Vegetarian diets, Mortality, Epidemiological study, Vegan, Pesco-vegetarian

## Abstract

The popularity of vegetarian diets has increased the need for studies on long-term health outcomes. A limited number of studies, including only one study from a non-vegetarian population, investigated the risk of mortality with self-identified vegetarianism and reported inconsistent results. This study evaluated prospective associations between vegetarian diets and all-cause mortality among 117,673 participants from the Prostate, Lung, Colorectal and Ovarian Cancer Screening Trial cohort study. Vegetarian diet status was self-identified on the questionnaire. Deaths were ascertained from follow-up questionnaires and the National Death Index database. Multivariable Cox regression models were used to estimate the risk of all-cause mortality in hazard ratio (HR) and 95% confidence intervals (CI). By diet group, there were 116,894 omnivores (whose diet does not exclude animal products), 329 lacto- and/or ovo-vegetarians (whose diet excludes meat, but includes dairy and/or eggs), 310 pesco-vegetarians (whose diet excludes meat except for fish and seafood) and 140 vegans (whose diet excludes all animal products). After an average follow-up of 18 years, 39,763 participants were deceased. The risk of all-cause mortality did not statistically significantly differ among the four diet groups. Comparing with the omnivore group, the HR (95% CI) were 0.81 (0.64–1.03) for pesco-vegetarian group, 0.99 (0.80–1.22) for lacto- and/or ovo-vegetarian group and 1.27 (0.99–1.63) for vegan group, respectively. Similarly, mortality risk did not differ when comparing lacto- and/or ovo-vegetarians plus vegans with meat/fish eaters (omnivores and pesco-vegetarians) (HR [95% CI] = 1.09 [0.93–1.28]). As this study is one of the two studies of vegetarianism and mortality in non-vegetarian populations, further investigation is warranted.

## Introduction

Chronic diseases including cardiovascular disease (CVD), cancer, and diabetes are the leading cause of deaths in the US [1]. Between 1990 and 2010, unhealthful diets accounted for 26% and 22% of all-cause and CVD deaths, respectively [2]. Vegetarian diets have gained popularity due to potential health, environmental and sustainability benefits [3].

Several foods of plant origin have been associated with a lower risk of chronic diseases and their risk factors such as insulin resistance and high total blood cholesterol [3–7]. These beneficial effects are linked to being often less energy-dense and more nutrient-rich with higher amounts of fiber, vitamins, minerals and phytochemicals and lower amounts of total and saturated fats compared with animal products [3]. Despite these health benefits, specific micronutrient deficiencies are of concern for vegans because animal products are the only natural source of vitamin B_12_ and provide more bioavailable iron and calcium [3].

Most previous observational studies and clinical trials lend support for protective effects of overall vegetarian diets or their specific components on risk of chronic diseases or their risk factors [5–7]. Few observational studies have investigated associations between vegetarian diets and all-cause mortality, and reported inconsistent associations [8–12]. Only one study was conducted in a general, non-vegetarian population in Australia and reported null association [11]. Hence, this study prospectively investigated whether self-identified vegetarian or vegan diets (i.e., lacto- and/or ovo-vegetarian, pesco-vegetarian, and vegan) compared with an omnivorous diet are associated with mortality risk in the US Prostate, Lung, Colorectal and Ovarian (PLCO) Cancer Screening Trial cohort.

## Methods

### Study population

This study was based on data from the PLCO Cancer Screening Trial which originally aimed to assess effectiveness of early cancer detection and to investigate etiological factors of cancer. Between 1993 and 2001, 154,952 participants (age range: 55 to 74) were enrolled and randomized into the control (standard cancer screening method) or intervention (new method) arm [13]. At the time of randomization, participants filled out questionnaires on possible risk factors for cancer including demographics, smoking history, medical history, body weight and height, and dietary intakes. Starting in 1998, a new food frequency questionnaire, Diet History Questionnaire (DHQ) [14], was administered within five years of randomization. This study focused on 118,779 participants who completed the DHQ. After excluding missing or unclear responses to questions on vegetarianism, 117,673 participants were included in the analysis. All participants included in this study provided written, informed consent for all trial activities and future etiological research studies. The PLCO study was approved by the Institutional Review Boards (IRB) at National Cancer Institute. The IRB review at Oregon State University was exempt.

### Dietary assessment and mortality ascertainment

The validated DHQ was self-administered and inquired about vegetarianism and habitual food and beverage intakes in the past 12 months [14]. Our analysis was based on questions about following any type of vegetarian diets and what foods they exclude from their diet entirely: meat (e.g., beef, pork, and lamb); poultry (e.g., chicken, turkey, and duck); fish and seafood; eggs; and dairy products (e.g., milk and cheese). The participants were classified into: (1) vegan (entirely excluding meat, poultry, fish and seafood, eggs and dairy products); (2) lacto- and/or ovo-vegetarian (entirely excluding meat, poultry, fish and seafood, but consuming dairy products and/or eggs); (3) pesco-vegetarian (entirely excluding meat, poultry, eggs and dairy products but consuming fish and seafood); and (4) omnivore (no exclusion of animal products). Deaths of participants were ascertained by annual study update questionnaires and regular linkage to the National Death Index database through 2018.

### Statistical analysis

Baseline characteristics were compared among the four diet groups using analysis of variance for continuous variables and χ^2^-test for categorical variables. For risk of mortality, we used the Cox regression and modeled on the time from the date of DHQ completion to the first of the following: date of death, loss to follow-up, or December 31, 2018. The hazard ratio (HR) and corresponding 95% confidence intervals (CI) were calculated first for pesco-vegetarian, lacto- and/or ovo-vegetarian or vegan diet group using the omnivore group as reference and second for vegetarians (i.e., lacto-and/or ovo-vegetarians and vegans) using non-vegetarians (i.e., pesco-vegetarians and omnivores [meat/fish eaters]) as reference. The following variables were *a priori* selected based on previous studies [8, 11, 15]: age, sex, study center location and trial arm (as part of the original PLCO trial) in the minimally-adjusted model. The fully-adjusted model also included smoking status, pack-years of smoking, alcohol consumption, race/ethnicity, education, body mass index (BMI) category and history of comorbidity at baseline. All covariates had no missing values except for BMI (N = 3486), pack-years of smoking (N = 3382) and education (N = 2308) and multiple imputation method was applied for these missing values. We conducted sensitivity analyses by excluding the first two years of the follow-up period. A two-sided test was performed, and the *p*-value used to test statistical significance was < 0.05.

## Results

Among 117,673 participants included in this study, 116,894 (99.3%) identified themselves as omnivores, 329 (0.3%) as lacto- and/or ovo-vegetarians, 310 (0.3%) as pesco-vegetarians, and 140 (0.1%) as vegans, respectively (Table 1). Participants in the omnivorous diet group were more likely to smoke cigarettes and drink alcohol and less likely to have completed college education than vegetarian or vegan groups. Furthermore, omnivores had the highest average BMI (27.2 kg/m^2^), followed by vegans (26.9 kg/m^2^), lacto- and/or ovo-vegetarians (25.7 kg/m^2^) and pesco-vegetarians (25.3 kg/m^2^). The proportions of non-Hispanic white participants ranged from 57.1% in the vegan diet group to 89.2% in the omnivore diet group.

**Table 1:** Baseline characteristics of study participants

	Omnivore	Lacto- and/or ovo-vegetarian	Pesco-vegetarian	Vegan
Number of participants	116,894	329	310	140
Age (years old)	65.7 ± 5.8	64.1 ± 5.5	64.4 ± 5.9	66.0 ± 6.4
Female	51.4%	57.2%	65.8%	48.2%
BMI (kg/m^2^)†	27.2 ± 4.8	25.7 ± 4.8	25.3 ± 4.6	26.9 ± 5.7
*Cigarette smoking status*				
Never smokers	48.3%	55.3%	58.7%	58.5%
Current smokers	9.3%	4.9%	2.3%	7.9%
Former smokers	42.4%	39.8%	39.0%	33.6%
Pack-years of smoking among smokers†	18.2 ± 27.1	12.0 ± 23.4	10.9 ± 20.6	13.5 ± 22.7
*Alcohol consumption*				
Non-drinkers	27.9%	52.3%	29.7%	49.3%
Moderate drinkers	59.4%	41.6%	57.4%	43.6%
Heavy drinkers	12.7%	6.1%	12.9%	7.1%
Race/ethnicity				
Non-Hispanic White	89.2%	65.1%	81.6%	57.1%
Other race/ethnicity	10.8%	34.9%	18.4%	42.9%
*Educational attainment†*				
High school or less	29.9%	17.6%	15.5%	30.9%
Some college	34.5%	26.2%	31.0%	30.9%
College or higher	35.6%	56.2%	53.5%	38.2%
Any comorbidity	38.9%	33.3%	30.2%	41.5%

The mean ± standard deviation for continuous variables and the proportion of participants (percentage) for categorical variables were included

Difference in characteristics among the four diet groups were tested by analysis of variance for continuous variables and χ^2^-test for categorical variables and all characteristics statistically significantly differed among the four diet groups with *P* < 0.05

^†^Those with missing values were excluded: BMI (N = 3486), pack-years of smoking (N = 3382) and education (N = 2308)

After an average follow-up of 18 years, 39,763 participants including 39,547 for omnivores, 88 for lacto- and/or ovo-vegetarian, 67 for pesco-vegetarians, and 61 for vegans were deceased (Table 2). After minimal adjustment (i.e., age, sex, study center locations and arms), comparing with the omnivore diet group, participants in the pesco-vegetarian group had a statistically significantly lower risk of mortality (HR [95% CI]: 0.67 [0.53–0.85]), but lacto- and/or ovo-vegetarian and vegan diet groups had no statistically significant difference in risk of mortality (HR [95% CI]: 0.88 [0.72–1.09] and 1.28 [0.99–1.64], respectively). After adjusting for all covariates, all three vegetarian diet groups had no statistically significant difference in risk of mortality. When comparing vegetarians (lacto- and/or ovo-vegetarian and vegans) with non-vegetarians (omnivores and pesco-vegetarians), risk of mortality also did not differ (HR [95% CI]: 1.01 [0.86–1.19] and 1.09 [0.93–1.28] after minimal and full adjustment, respectively). These association patterns were unchanged after excluding the first two years of the follow-up period (data not shown).

**Table 2:** Risk of all-cause mortality by vegetarian diet groups

	Omnivore	Pesco-vegetarian	Lacto- and/or ovo-vegetarian	Vegan	*P-difference*
The number of deaths	39,547	67	88	61	–
Minimally adjusted HRs (95% CI) for the four diet groups	Reference	0.67 (0.53–0.85)	0.88 (0.72–1.09)	1.28 (0.99–1.64)	0.001
Fully-adjusted HR (95% CI) for the four diet groups	Reference	0.81 (0.64–1.03)	0.99 (0.81–1.22)	1.27 (0.99–1.63)	0.09
Minimally adjusted HRs (95% CI) for vegetarians vs non-vegetarians	Reference		1.01 (0.86–1.19)		0.90
Fully-adjusted HR (95% CI) for vegetarians vs non-vegetarians	Reference		1.09 (0.93–1.28)		0.31

Minimally adjusted models include age (continuous), sex (male or female), study center location, trial arm (control vs intervention as part of original PLCO trial) and fully-adjusted models also includes smoking status (never, former or current), pack-years of smoking (never smokers, ≤ 10, 10–20 or ≥ 20 pack-years of smoking), alcohol consumption (non-drinkers, moderate or high drinkers), non-Hispanic white race/ethnicity and educational attainment (high school or less, some college, or college or higher), BMI (according to the World Health Organization; underweight, normal weight, overweight or obesity) category and history of comorbidity any one of stroke, heart attack, hypertension and/or diabetes, or none) at baseline

## Discussion

In this prospective analysis of a previous cancer-screening cohort of middle-aged and older adults in the US, over 99% of the participants self-identified as non-vegetarian and the rest followed lacto- and/or ovo-vegetarian, pesco-vegetarian or vegan diets. Pesco-vegetarians had a lower mortality risk than omnivores and vegans, which lost statistical significance after further adjusting for lifestyle and sociodemographic factors and comorbidity. We observed no difference in risk of all-cause mortality between vegetarian and non-vegetarian diets.

Previously, observational studies reported that vegetarian diets were associated with a lower risk of chronic diseases (e.g., CVD and type 2 diabetes) and their risk factors (e.g., obesity, blood lipid profiles and glucose control), compared with omnivorous diets; however, findings were less clear in terms of all-cause mortality [3, 4]. Comparing with omnivores, no difference in risk of mortality among vegans and lacto- and/or ovo-vegetarians were reported in all three previous studies [8, 11, 12]. For pesco-vegetarians, comparing with omnivores, the Adventist Health Study 2 reported a statistically significant inverse association (0.81 [0.69–0.94]) [12], but not the other study (0.79 [0.59–1.06]) [11]. Although the comparison was with regular meat eaters, instead of omnivores as in our and other previous studies, in the Oxford Vegetarian/Oxford- European Prospective Investigation into Cancer and Nutrition (EPIC) Study, the risk of mortality did not differ from vegans, lacto- and/or ovo-vegetarians or pesco-vegetarians [8]. Furthermore, previous six studies compared risk of mortality between vegetarians and non-vegetarians. Three studies of Seventh-Day Adventists—the Adventist Mortality Study and Adventist Health Study 1 and 2—reported an inverse association [12, 16], but other three previous studies reported no significant association [9–11]. Altogether, our null finding is consistent with the previous studies of vegetarianism and all-cause mortality, except for cohort studies of Adventists.

Differences in associations may be due to the fact that self-identified vegans in our study might have differed in chronic diseases and their risk factors from those of previous studies targeting vegetarians such as Adventist studies. The high prevalence of comorbidities and BMI in the vegan group compared with the Adventist and other previous studies, in which vegans had the lowest average BMI among the diet groups [12, 17], may indicate that our self-identified vegans recently switched to a vegan diet because of health concerns. In our questionnaire, vegetarianism questions only pertained to the past 12 months, lacking the information on the specific duration of following vegetarian/vegan diets. In the previous pooled analysis of five prospective studies, participants who have followed a vegetarian diet for > 5 years had a lower risk of all-cause mortality than omnivores (0.93 [0.79–1.09]), but those followed ≤ 5 years had an increased risk (1.20 [1.04–1.38]) [16]. In addition, smoking and alcohol drinking were far more common in our study than Adventist Health Study 2 (9.3% vs. 1.1% for current smokers and 72.0% vs 10.1% for alcohol drinkers, respectively). Given that the PLCO study was conducted in a general, non-vegetarian population and same association patterns persisted after excluding the first two years of the follow-up period, the potentially shorter duration of following vegetarian/vegan diets might not have been sufficiently long enough for our study participants to have the typical health benefits associated with vegetarian/vegan diets.

Strength of this study is its prospective collection of diet and other important confounders with a relatively long follow-up (18 years) of death and vital status. In addition, our study is the first study conducted in a general, non-vegetarian population in the US to investigate associations between vegetarianism and mortality. A potential limitation, however, is low proportions of vegans/vegetarians in our study population (0.7%), which was lower than a previous report (1.9%) [18], but higher than another (0.4%) [19]. In addition, the self-identified vegetarian diet status was based on two questions regarding foods they excluded from their diet. Because of this, a possibility of misclassifications for each diet group cannot be fully ruled out, as reported previously on discrepancies between self-identified vegetarianism and their food and beverage consumption [20]. Classifying vegetarian diet status based on food and beverage consumption is beyond the scope of this study and warrants further investigation. Furthermore, we did not have information on the duration of vegetarian diets, which was reported to modify the direction of associations with mortality [16]. Due to the very small number of deaths in vegans and vegetarians, we were unable to conduct cause-specific nor subgroup analyses. Lastly, physical activity information at baseline was not collected and we were unable to adjust in our analyses.

In conclusion, we found no difference in risk of all-cause mortality by self-identified vegetarian diet status. Similar to previous studies, our vegetarians attained more education, were less likely to smoke cigarettes and drink alcohol, and had a lower BMI; however, vegans had higher comorbidities and similar BMI to omnivorous participants, suggesting that they likely recently switched to vegans because of health concerns. Future studies of vegetarian diets and mortality need to be conducted in general populations and consider the duration of following vegetarian diets.

## Abbreviations

BMI: Body mass index
CVD: Cardiovascular disease
DHQ: Diet History Questionnaire
EPIC: European Prospective Investigation into Cancer and Nutrition study
PLCO: Prostate, Lung, Colorectal and Ovarian Cancer Screening Trial study
